# The association between metabolic syndrome and heart failure in middle-aged male and female: Korean population-based study of 2 million individuals

**DOI:** 10.4178/epih.e2022078

**Published:** 2022-09-21

**Authors:** Tae-Eun Kim, Hyeongsu Kim, JiDong Sung, Duk-Kyung Kim, Myoung-Soon Lee, Seong Woo Han, Hyun-Joong Kim, Sung Hea Kim, Kyu-Hyung Ryu

**Affiliations:** 1 Department of Clinical Pharmacology, Konkuk University Medical Center, Seoul, Korea; 2 Department of Preventive Medicine, Konkuk University School of Medicine, Seoul, Korea; 3 Division of Cardiology, Department of Medicine, Heart Vascular Stroke Institute, Samsung Medical Center, Sungkyunkwan University School of Medicine, Seoul, Korea; 4 Department of Social and Preventive Medicine, Sungkyunkwan University School of Medicine, Suwon, Korea; 5 Division of Cardiology, Dongtan Sacred Heart Hospital, Hallym University College of Medicine, Hwaseong, Korea; 6 Division of Cardiology, Department of Internal Medicine, Konkuk University Medical Center, Konkuk University School of Medicine, Seoul, Korea

**Keywords:** Metabolic syndrome, Heart failure, Big data, Sex difference

## Abstract

**OBJECTIVES:**

Although an association is known to exist between metabolic syndrome (MetS) and heart failure (HF) risk, large longitudinal studies are limited. We investigated metabolic status as a risk factor for HF in middle-aged male and female and considered sex differences in various risk factors for HF using nationwide real-world data.

**METHODS:**

Data obtained from the Korean National Health Insurance Service from 2009 to 2016 were analyzed. A total of 2,151,597 middle-aged subjects (between 50 and 59 years old) were enrolled. Subjects were divided into 3 groups (normal, pre-MetS, and MetS). Cox proportional hazard models were used to estimate the association between MetS and incident HF after adjusting for clinical risk factors.

**RESULTS:**

At baseline, MetS existed in 23.77% of male and 10.58% of female. Pre-MetS and MetS increased the risk of HF: the hazard ratios of pre-MetS for incident HF were 1.508 (95% confidence interval [CI], 1.287 to 1.767) in male and 1.395 (95% CI, 1.158 to 1.681) in female, and those of MetS were 1.711 (95% CI, 1.433 to 2.044) in male and 2.144 (95% CI, 1.674 to 2.747) in female. Current smoking, a low hemoglobin level, underweight (body mass index <18.5 kg/m^2^), a high creatinine level, and acute myocardial infarction were also predictors of HF in both sexes.

**CONCLUSIONS:**

Pre-MetS and MetS were identified as risk factors for HF in middle-aged male and female. The effect of MetS on the occurrence of HF was stronger in female than in male. Pre-MetS was also a predictor of HF, but was associated with a lower risk than MetS.

## INTRODUCTION

Heart failure (HF) is a clinical syndrome with symptoms and/or signs caused by a structural and/or functional cardiac abnormality and corroborated by elevated natriuretic peptide levels and/or objective evidence of pulmonary or systemic congestion [1]. Unlike other cardiac disorders, the prognoses of which have markedly improved, only modest survival improvement has been observed in HF patients, and the prevalence of HF is rising [2–4].

Metabolic syndrome (MetS), a cluster of cardiovascular risk factors that includes hypertension (HTN), central obesity, insulin resistance, and atherogenic dyslipidemia, is common worldwide. The prevalence of MetS in males and females is 35.6% and 30.3% in the United States [5] and 41% and 38% in Europe [6]. Although the pathophysiologic mechanism of MetS has not been fully elucidated, obesity and insulin resistance are believed to play a critical role in the pathogenesis of MetS [7].

Many studies have demonstrated an association between HF and MetS. Miura et al. [8] reported that the prevalence of MetS was 2-fold higher in patients with HF than in the general population, and Li et al. [9] found that MetS was associated with an approximately 2-fold increased likelihood of self-reported HF in a population-based cross-sectional study. Various studies have also reported associations between MetS and the prognosis of HF [10,11]. Although the underlying pathophysiologic mechanisms of the association between MetS and HF remain to be fully elucidated, metabolic stress caused by elevated glucose and free fatty acids, including dysregulated insulin signaling, impaired mitochondrial respiration, and reactive oxygen species formation, is thought to decrease adenosine triphosphate (ATP) production, resulting in impaired contraction, myocellular hypertrophy, and fibrosis of the heart, subsequently leading to HF [12].

Despite extensive evidence showing the association between MetS and HF, there are few longitudinal studies that allow causal inferences, and the majority of them were carried out among elderly people. Even though a longitudinal study in middle-aged individuals was published, only males were involved [13]. The authors hypothesized that the risk of MetS for HF may be different by age group as well as by sex. Thus, in this study, we investigated whether MetS is a predictor of HF in both males and females in their 50s using the National Health Insurance Service (NHIS) database. We also investigated the difference in the degree of the risk of MetS associated with HF between males and females.

## MATERIALS AND METHODS

### Database source

This study used the database of the NHIS, which is the universal health insurance system in Korea. The NHIS covers more than 97% of the population, and its database contains information on patients’ demographics (age, sex, socioeconomic variables, etc.), prescribed drugs (generic drug name, prescription date, and the duration and route of administration) and use of medical care institutions (hospital admissions, outpatient department visits, pharmaceutical visits, etc.). The NHIS also provides an annual or biennial health screening examination called the National Health Screening Program to the population aged 20 years and older, which includes questionnaires on lifestyle and behavior, physical examinations, and blood tests. In the NHIS database, diagnoses are coded according to the International Classification of Diseases-10th revision (ICD-10). The data were provided by the NHIS after de-identification.

### Study population

**Figure 1:**
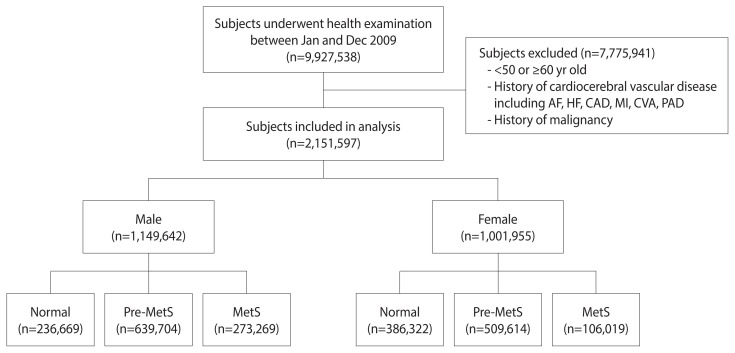
Flow diagram of the study population. Pre-MetS, pre-metabolic syndrome; MetS, metabolic syndrome; AF, atrial fibrillation; HF, heart failure; CAD, coronary artery disease; MI, myocardial infarction; CVA, cerebrovascular accident; PAD, peripheral arterial disease.

The number of individuals who underwent health screening examinations between January and December 2009 in Korea was 9,927,538. Among these 9,927,538 participants, 7,775,941 were excluded for the following reasons: (1) age <50 years old or ≥60 years old; and (2) history of malignancy (ICD-10 codes C00.X-C99.X) or (3) history of cardiovascular or cerebrovascular disease, including atrial fibrillation (ICD-10 codes I48), coronary artery disease (procedure codes M6561-4), myocardial infarction (ICD-10 codes I21), HF (ICD-10 codes I42 or I50), cerebrovascular accident (ICD-10 codes I60.X-I609.X), and peripheral arterial disease (ICD-10 codes I73 or I74) within 5 years from the screening. HF was considered to have been diagnosed when the diagnostic code of HF occurred and there was any history of admission to the hospital. The final number of participants in the study was 2,151,597. These subjects were then divided into 3 groups for each sex according to the number of MetS components: normal group (0), pre-MetS group (1–2), and MetS group (3–5). We analyzed follow-up data until December 31, 2016 (Figure 1).

### Definition of metabolic syndrome

According to the modified criteria of the National Cholesterol Education Program/Adult Treatment Panel III criteria [14], a diagnosis of MetS was made when at least 3 of the following 5 components were present: (1) abdominal obesity (waist circumference ≥90 cm for males, ≥85 cm for females); (2) elevated blood pressure (systolic blood pressure ≥130 mmHg, diastolic blood pressure ≥85 mmHg, or treatment of previously diagnosed HTN); (3) elevated fasting glucose (≥100 mg/dL or treatment of previously diagnosed diabetes mellitus [DM]); (4) high triglycerides (TGs; ≥150 mg/dL or drug treatment for high TGs); and (5) low high-density lipoprotein cholesterol (HDL-C; <40 mg/dL for males, <50 mg/dL for females or drug treatment for low HDL-C). Subjects with 1 or 2 MetS components were defined as having pre-MetS, and those with no MetS components were defined as normal.

### Primary outcome and follow-up

The primary outcome of this analysis was the incidence of HF during the follow-up period. We defined an HF event using newly occurring ICD-10 codes for HF (I50). Follow-up was initiated at the date of the health screening examination and ended at the incidence of HF, death, or December 31, 2016, whichever came first.

### Statistical analysis

Descriptive statistics were used to present the characteristics of the study subjects. The comparisons of baseline characteristics among subjects with different MetS statuses were performed with the chi-square test.

Cox proportional hazard models were used to estimate hazard ratios (HRs) and 95% confidence intervals (CIs) for the incidence of HF during the follow-up period. Before using the Cox proportional hazard model, log-log survival curves were plotted to test the proportional hazard assumption. The models were initially unadjusted, and further adjustments were made for demographic characteristics (age, smoking status, and exercise status) (model 1). Model 2 was adjusted for the same covariates as model 1, as well as family history of HTN, stroke, and DM. Covariates were added to those of previous models step by step; thus, model 3 was adjusted for body mass index (BMI) and laboratory results (hemoglobin, creatinine, total cholesterol, low-density lipoprotein cholesterol, and alanine aminotransferase [ALT]) as well as demographic characteristics and family history. Model 4 was adjusted for demographic characteristics, family history, BMI, laboratory results, and the occurrence of acute myocardial infarction (AMI) during the follow-up period. To examine the risk of HF per individual component of Mets, additional analysis was performed with final model, in which the 5 components of MetS (abdominal obesity, elevated blood pressure, elevated fasting glucose, high TG, low HDL-C) were used instead of metabolic status.

All tests were 2-sided, with a significance level of 0.05. All analyses were conducted using SAS version 9.4 (SAS Institute Inc., Cary, NC, USA).

### Ethics statement

This study was approved by the NHIS of Korea (No. NHIS-2020-1-153) and the Institutional Review Board of Konkuk University Medical Center (No. KUH 2020-07-096).

## RESULTS

### Baseline characteristics

A total of 1,149,642 males and 1,001,955 females were included in this analysis. At baseline, MetS existed in 23.77% of males and 10.58% of females. The prevalence of pre-MetS was 55.64% and 50.86% in males and females, respectively. Table 1 presents baseline characteristics by MetS status. The prevalence of MetS was significantly associated with smoking status, alcohol consumption, and the frequency of exercise. The prevalence of MetS also exhibited significant associations with family history of HTN, DM, and stroke; BMI; and the clinical laboratory results of total cholesterol, hemoglobin, creatinine, and ALT in both sexes (Table 1).

### Association between metabolic syndrome status and heart failure

**Figure 2:**
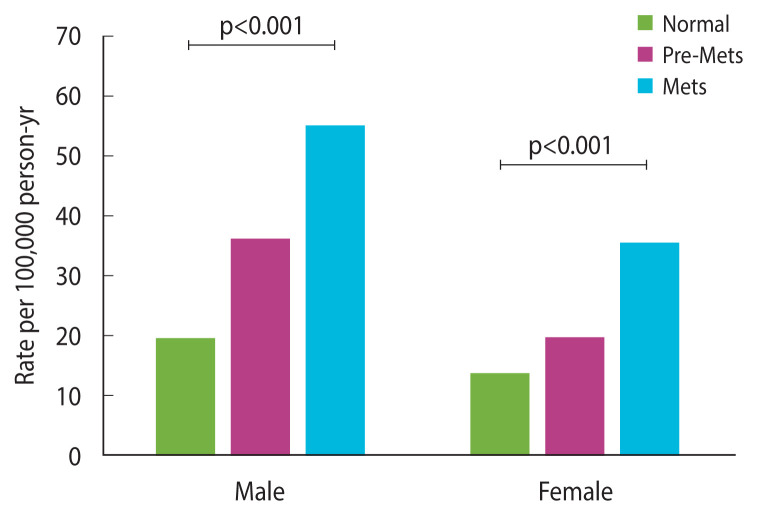
Incidence rate of heart failure. Pre-MetS, pre-metabolic syndrome; MetS, metabolic syndrome.

The incidence rate of HF was associated with MetS status. The incidence increased as pre-MetS progressed to MetS and was higher in males than in females. The rates per 100,000 person-years in the normal population were 19.20 in males and 13.38 in females, and the rates in the pre-MetS and MetS populations were 35.88 and 55.05, respectively, in males and 19.42 and 34.91, respectively, in females (Figure 2).

Table 2 compares the baseline characteristics of the population with HF and those without HF. Both in males and females, the frequency of HF was higher in the population with MetS than in the normal population. The pre-MetS population also showed a higher frequency of HF than the normal population, although the proportion was lower than that of the MetS group.

In addition to MetS status, statistically significant differences were found in baseline characteristics between populations with and without HF. In both males and females, current smokers showed a higher proportion of HF incidence than ex-smokers or non-smokers. The occurrence of AMI during the follow-up period was clearly related to a higher frequency of HF. In comparison to the normal BMI group, both the underweight and overweight (obese) groups showed higher HF frequency in both sexes. Laboratory findings including cholesterol, creatinine, and ALT and a family history of HTN were also related to the frequency of HF in both sexes. Differences in HF frequency according to alcohol consumption, frequency of exercise, family history of DM, and hemoglobin level were shown only in males (Table 2).

### Risk factors for heart failure

Multivariable Cox regression analysis was performed to evaluate risk factors for HF (Tables 3 and 4). Without other covariates, the HRs of pre-MetS and MetS for HF were 1.867 (95% CI, 1.599 to 2.180) and 3.016 (95% CI, 2.569 to 3.541), respectively, in males and 1.605 (95% CI, 1.340 to 1.923) and 3.349 (95% CI, 2.706 to 4.145), respectively, in females (model 1). Considering age; smoking; alcohol consumption; exercise; family history of heart disease, HTN, DM, and stroke; laboratory results; and the occurrence of AMI during the follow-up period as covariates, the occurrence of HF was still significantly higher in subjects with pre-MetS or MetS than in normal individuals, with higher HRs observed in MetS patients than pre-MetS patients. The risk of HF associated with pre-MetS was slightly higher in males than in females, whereas that associated with MetS was higher in females than in males (HR of pre-MetS: 1.508; 95% CI, 1.287 to 1.767 in males and 1.395; 95% CI, 1.158 to 1.681 in females; HR of MetS: 1.711; 95% CI, 1.433 to 2.044 in males and 2.144; 95% CI, 1.674 to 2.747 in females) (model 5; Tables 3 and 4).

In addition, the occurrence of AMI during the follow-up period was associated with a 131-fold and 83-fold higher risk for HF in males and females, respectively. Although the study participants were all in their 50s, the risk of HF increased with increasing age. Current smoking increased the risk of HF by 1.2-fold (HR,1.190; 95% CI, 1.051 to 1.348) in males and 1.7-fold in females (HR, 1.721; 95% CI, 1.254 to 2.362). Low hemoglobin levels and elevated creatinine levels were also risk factors for HF. The HR of low hemoglobin was 1.553 (95% CI, 1.319 to 1.829) in males and 1.203 (95% CI, 1.006 to 1.439) in females; the HR (95% CI) of elevated creatinine level was 1.624 (95% CI, 1.362 to 1.937) in males and 2.167 (95% CI, 1.510 to 3.109) in females.

BMI as a risk factor for HF showed different results by sex. Overweight was a significant risk factor for HF in females with obesity (BMI ≥30.0 kg/m^2^), increasing the risk by 2-fold (HR, 2.085; 95% CI, 1.523 to 2.853), whereas overweight was not a risk factor for HF in males. Underweight (BMI <18.5 kg/m^2^) increased the risk of HF in males and females by 1.8-fold (HR, 1.786; 95% CI, 1.266 to 2.520) and 1.7-fold (HR, 1.663; 95% CI, 1.053 to 2.624), respectively.

Alcohol consumption, total cholesterol and ALT levels exhibited inconsistent relationships with the occurrence of HF by sex. Only in males, the consumption of 2–3 servings of alcohol per month and a slight elevation of total cholesterol (200–239 mg/dL) decreased the risk of HF by 19% (HR, 0.814; 95% CI, 0.729 to 0.909) and 11% (HR, 0.888; 95% CI, 0.799 to 0.986) respectively. A slight elevation of the ALT level (40–99 IU/L) increased the risk of HF (HR, 1.551; 95% CI, 1.193 to 2.016) only in females, and an elevation of the ALT level over 100 IU/L) increased the risk of HF (HR, 1.591; 95% CI, 1.183 to 2.139) only in males.

In the investigation of the risk of HF for each component of MetS, elevated blood pressure and elevated fasting glucose increased the risk of HF both in males and females by 1.17–1.37 fold. Abdominal obesity was only the risk factor for males. High TG and low HDL-C levels did not show significant effects (Table 5).

## DISCUSSION

Associations between the prevalence or prognosis of HF and MetS have been shown in many studies [8–11]. However, most of these studies were cross-sectional in design, which prevents drawing causal inferences, and only a few longitudinal studies have been reported. A longitudinal study with 2,314 middle-aged males reported that MetS was a significant risk factor for HF, with an HR of 1.80 (95% CI, 1.11 to 2.91) [13]. A study of elderly individuals in their 70s reported that MetS increased the occurrence of HF, with an HR of 1.49 (95% CI, 1.10 to 2.00) [15], and another study of elderly individuals with a mean age of 69 years also reported that MetS was a predictor of HF, with an HR of 1.58 (95% CI, 1.16 to 2.15) [16]. In contrast, in a study of participants with a mean age of 62 years, MetS was not a significant risk factor for HF [14]. The present study was a large population-based study that included over 2 million individuals in their 50s. MetS was a significant risk factor for HF, and the HR in males was similar to that reported in a previous study of middle-aged males [13]. Although the results of studies in elderly populations are inconsistent, the HR in the present study was higher than that in a study conducted among elderly participants in which MetS was reported to be a significant risk factor for HF [15,16].

The present study revealed that the effect of MetS on HF differs by sex. The HR in males was 1.711 (95% CI, 1.433 to 2.044) and that in females was 2.144 (95% CI, 1.674 to 2.747), showing a higher risk of MetS associated with HF in females than in males. The authors speculate that this sex difference in the risk of MetS for HF is due to the difference in the distribution of HF subtypes between males and females. It was reported that patients with HF with preserved ejection fraction (HFpEF) are twice as likely to be females, while males have a 2-fold higher cumulative incidence of HF with reduced ejection fraction (HFrEF) than HFpEF [17–19]. Both in HFrEF and HFpEF, inappropriate or excessive inflammation is a major factor damaging the heart; however, the cause of the inflammation differs. HFpEF is associated with inflammation brought on by a cluster of metabolic risk factors such as obesity, diabetes, and HTN, whereas HFrEF is associated with sterile inflammation induced by myocardial infarction or toxic necrosis, or non-sterile inflammation induced by viral infection [20]. Given this knowledge, despite not discriminating between HFrEF and HFpEF in this study, the authors hypothesize that the proportion of HFpEF was likely higher in females than in males, and as a result, the risk of MetS for HF was evaluated to be higher in females than in males. This theory is limited, however, by the fact that the risk of pre-MetS for HF was marginally higher in males, whose predominant subtype is HFrEF. Further research is needed on the mechanism underlying sex differences in the risk of MetS.

In the additional investigation of the risk of HF per individual MetS component, 3 of 5 components (abdominal obesity, elevated blood pressure, and elevated fasting glucose) increased the risk of HF. Interestingly, none of the HRs of those 3 components were greater than those of MetS or pre-MetS. This means that individuals diagnosed with MetS or pre-MetS have a higher risk for HF than those who have only 1 component of MetS.

In this study, we considered the known risk factors for HF as covariates for adjustment in the Cox regression analysis. It is well known that AMI is a major risk factor for HF. In a study with a median follow-up time of 3.2 years, 31% of males and 46% of females developed HF among those hospitalized due to AMI [21]. Another study reported that approximately 84% of patients with coronary heart disease developed HF during a 19-year follow-up [22]. In the present study, AMI increased the risk of HF by 131-fold in males and 83-fold in females, which are considerably higher risks than those previously reported. In a study using data from the National Health and Nutrition Examination Survey [22], the relative risk of AMI was 8, and in the Framingham study [23], the HR was 6. The reason that the HR of AMI was extremely high in our study is thought to be that the study population was composed of relatively healthy individuals, and patients with a history of cardiocerebrovascular diseases were excluded; additionally, the follow-up period was relatively short compared to that of the NHANES study or the Framingham study. Namely, in people who do not have an underlying disease that can lead to HF, the effect of AMI on the occurrence of HF within a short period is suspected to be critical. The HR of AMI for HF was higher in males than in females, coinciding with the findings of a previous report [22].

The association between BMI and HF exhibited obvious sex differences. In females, BMI of 23.0–29.9 kg/m^2^ increased the risk of HF by 1.2-fold to 1.3-fold, and obesity (BMI ≥30.0 kg/m^2^) increased it by 2.1-fold, whereas in males, overweight was not a significant risk factor for HF. This result is consistent with many studies that reported that overweight increased the HF risk in females more significantly than in males [22,24–26]. In the present study, underweight (BMI <18.5 kg/m^2^) increased the risk of HF in both males and females. Although various studies have reported associations between BMI and HF, underweight has rarely been separately evaluated [27–29]. The increased risk of HF by underweight is supported by a recent study that reported associations among obesity degree, glycemic status, and risk of HF [30].

In this study, low hemoglobin was a risk factor for HF; however, high hemoglobin levels were not a risk factor despite the significantly high prevalence of HF in individuals with high hemoglobin levels. Low hemoglobin levels, lower blood viscosity, hypoxia, and enhanced nitric oxide activity induce reduced vascular resistance, followed by increased cardiac output. Increased cardiac output leads to left ventricular hypertrophy and cardiac enlargement, which can eventually lead to HF [31]. Different results have been reported regarding the association between HF and hemoglobin levels; while Klip et al. [32] reported that both low levels and high levels of hemoglobin increased the risk of HF compared to the risk associated with normal hemoglobin levels, Coglianese et al. [33] reported that high and normal hematocrit levels were associated with a higher risk of HF than low hematocrit levels. These reports explained that a high level of hemoglobin increases vascular resistance by scavenging nitric oxide, a vasodilator, which induces hypertension, left ventricular hypertrophy, and finally HF. These different associations between hemoglobin level and HF risk are suspected to result from the different follow-up periods and different study populations. While the follow-up period of our study was relatively short, with a maximum of 8 years, the study of hematocrit and HF [33] was performed over 20 years, and the study of hemoglobin and HF had a median follow-up of 6 years [32]. Additionally, while our study was performed in a healthy population without underlying cardiovascular disease, which is closely related to HF, the study of hemoglobin and HF [32] did not exclude participants with those underlying diseases.

The findings of this study, which indicated that MetS is a risk factor for HF in both males and females, have substantial implications since they indicate that preventing MetS can help avoid HF. The necessity of early management is further emphasized by the finding that both MetS and pre-MetS were risk factors for HF. Notably, despite HF being less common in females, females had a higher risk of developing HF via MetS than males did. This shows that even if females are more rarely affected by MetS than males are, attention should be paid to MetS in females.

Despite the positive aspects of the present study, there were some limitations that should be addressed. First, this study evaluated the subjects’ metabolic status based on data from 2009. Even if a subject who had been classified into the normal group on the basis of the data from 2009 developed MetS during the follow-up period, the subject was analyzed in the normal group. Although the number of subjects whose metabolic status changed may not have been substantial considering the total study population, this issue could nonetheless affect the accuracy of the results. Second, we were unable to distinguish between HFrEF and HFpEF due to the lack of ejection fraction information in the NHIS data. Since the clinical characteristics and pathophysiology of HFrEF and HFpEF are different, the impact of MetS may be different in these 2 HF subtypes. Furthermore, our finding that the risk of MetS for HF varied by sex is likely explained by the different distribution of HFrEF and HFpEF between males and females. Third, we defined an HF event using newly occurring ICD-10 codes for HF. The number of patients based on ICD-10 codes may be higher or lower than the actual number, because the NHIS database was not collected for research purposes. Finally, our study excluded subjects with a history of malignancy or cardiocerebral vascular disease; thus, the association between MetS status and HF risk was analyzed in a relatively healthy population, which means that these results may not be generalizable to high-risk populations.

In summary, this study evaluated the association between MetS and the risk of HF in middle-aged males and females using nationwide real-world data. MetS was a risk factor for HF, and its effect on HF was stronger in females than in males. Pre-MetS was also a predictor of HF, but pre-MetS was associated with a lower risk than MetS.

## Figures and Tables

**Table 1.** Baseline characteristics of the study population according to MetS status

Characteristics		Male				Female			
		Normal	Pre‐MetS	MetS	p-value^1^	Normal	Pre‐MetS	MetS	p-value^1^
Total		236,669 (20.59)	639,704 (55.64)	273,269 (23.77)		386,322 (38.56)	509,614 (50.86)	106,019 (10.58)	
Smoking status					<0.001				<0.001
	Non-smoker	69,695 (23.23)	166,871 (55.62)	63,454 (21.15)		367,335 (38.69)	483,018 (50.87)	99,076 (10.44)	
	Ex-smoker	61,530 (19.97)	172,740 (56.08)	73,774 (23.95)		5,641 (39.69)	6,929 (48.75)	1,642 (11.55)	
	Current smoker	103,969 (19.42)	296,719 (55.43)	134,594 (25.14)		10,503 (33.77)	16,075 (51.68)	4,525 (14.55)	
Alcohol consumption					<0.001				<0.001
	None	76,042 (24.28)	172,399 (55.04)	64,770 (20.68)		270,576 (38.33)	359,005 (50.85)	76,363 (10.82)	
	2-3 drinks/mo	114,928 (20.92)	305,899 (55.68)	128,574 (23.40)		94,636 (39.97)	119,561 (50.50)	22,545 (9.52)	
	1-4 drinks/wk	32,605 (15.79)	116,225 (56.28)	57,695 (27.94)		11,672 (34.71)	17,863 (53.13)	4,089 (12.16)	
	≥5 drinks/wk	10,238 (14.87)	38,795 (56.36)	19,803 (28.77)		3,835 (33.26)	6,098 (52.89)	1,597 (13.85)	
Exercise (times/wk)					<0.001				<0.001
	No exercise	93,741 (20.12)	257,668 (55.30)	114,561 (24.59)		193,655 (37.68)	263,109 (51.19)	57,202 (11.13)	
	1-4	47,105 (20.44)	127,964 (55.53)	55,388 (24.03)		71,043 (39.03)	92,188 (50.65)	18,771 (10.31)	
	≥5	93,704 (21.11)	248,941 (56.08)	101,220 (22.80)		119,010 (39.76)	150,914 (50.43)	29,360 (9.81)	
Family history of hypertension					<0.001				<0.001
	Yes	18,324 (15.33)	65,336 (54.64)	35,905 (30.03)		40,687 (31.85)	68,343 (53.50)	18,707 (14.64)	
	No	145,852 (21.44)	378,540 (55.63)	156,040 (22.93)		213,678 (39.88)	268,648 (50.14)	53,509 (9.99)	
Family history of diabetes mellitus					<0.001				<0.001
	Yes	17,843 (16.18)	59,802 (54.24)	32,617 (29.58)		34,730 (33.88)	52,863 (51.57)	14,922 (14.56)	
	No	146,165 (21.21)	383,657 (55.68)	159,236 (23.11)		219,524 (39.15)	283,972 (50.64)	57,220 (10.20)	
Family history of stroke					<0.001				<0.001
	Yes	16,413 (19.26)	47,273 (55.47)	21,534 (25.27)		23,950 (36.59)	33,966 (51.90)	7,533 (11.51)	
	No	47,711 (20.67)	396,629 (55.49)	170,413 (23.84)		230,167 (38.53)	302,655 (50.67)	64,531 (10.80)	
Total cholesterol (mg/dL)					<0.001				<0.001
	<200	151,035 (25.05)	332,175 (55.09)	119,760 (19.86)		245,784 (41.12)	301,128 (50.38)	50,829 (8.50)	
	200-239	70,069 (17.51)	226,451 (56.58)	103,697 (25.91)		114,051 (37.05)	156,365 (50.80)	37,389 (12.15)	
	>239	15,565 (10.63)	81,078 (55.36)	49,812 (34.01)		26,487 (27.47)	52,121 (54.06)	17,801 (18.46)	
ALT (IU/L)					<0.001				<0.001
	<40	216,861 (23.54)	526,325 (57.13)	178,156 (19.34)		376,523 (39.49)	485,460 (50.91)	91,557 (9.60)	
	40-99	18,121 (8.60)	105,617 (50.10)	87,087 (41.31)		8,696 (19.87)	21,857 (49.95)	13,209 (30.18)	
	≥100	1,687 (9.65)	7,762 (44.42)	8,026 (45.93)		1,103 (23.71)	2,297 (49.37)	1,253 (26.93)	
Hemoglobin (g/dL)					<0.001				<0.001
	<13.5 (male), <12.0 (female)	23,156 (26.50)	48,700 (55.74)	15,510 (17.75)		90,985 (40.86)	112,912 (50.70)	18,792 (8.44)	
	13.5-17.5 (male), 12.0-15.5 (female)	212,536 (20.25)	584,907 (55.73)	252,155 (24.02)		13,166 (37.99)	394,040 (50.89)	86,041 (11.11)	
	>17.5 (male), >15.5 (female)	977 (7.71)	6,097 (48.09)	5,604 (44.20)		1,151 (23.30)	2,619 (53.02)	1,170 (23.68)	
Creatinine (mg/dL)					<0.001				<0.001
	≤1.5	228,987 (20.67)	616,190 (55.63)	262,468 (23.70)		381,018 (38.59)	501,914 (50.84)	104,342 (10.57)	
	>1.5	7,660 (18.27)	23,478 (56.00)	10,788 (25.73)		5,281 (36.11)	7,671 (52.45)	1,674 (11.45)	
Body mass index (kg/m^2^)					<0.001				<0.001
	<18.5	8,184 (48.01)	8,264 (48.48)	599 (3.51)		15,574 (61.25)	9,535 (37.50)	319 (1.25)	
	18.5-22.9	123,762 (34.53)	205,799 (57.42)	28,877 (8.06)		243,305 (49.84)	227,833 (46.67)	17,019 (3.49)	
	23-24.9	68,418 (21.00)	201,763 (61.92)	55,641 (17.08)		85,685 (35.64)	133,579 (55.57)	21,128 (8.79)	
	25-29.9	36,122 (8.74)	214,726 (51.95)	162,486 (39.31)		41,084 (18.76)	126,189 (57.63)	51,685 (23.60)	
	≥30.0	183 (0.52)	9,152 (26.15)	25,666 (73.33)		674 (2.320)	12,478 (43.00)	15,868 (54.68)	

Values are presented as number (%). MetS, metabolic syndrome; Pre-MetS, pre-metabolic syndrome; ALT, alanine aminotransferase.

1 Using the chi-square test.

**Table 2.** Baseline characteristics of study population according to HF

Characteristics		Male			Female		
		Non-HF	HF	p-value^1^	Non-HF	HF	p-value^1^
Metabolic status							
	Normal	236,373 (99.87)	296 (0.13)	<0.001	386,025 (99.92)	297 (0.08)	<0.001
	Pre‐MetS	638,221 (99.77)	1,483 (0.23)		509,001(99.88)	612 (0.12)	
	MetS	272,285 (99.64)	984 (0.36)		105,769 (99.76)	250 (0.24)	
Smoking status				<0.001			<0.001
	Non-smoker	299,458 (99.81)	562 (0.19)		948,372 (99.89)	1,056 (0.11)	
	Ex-smoker	307,501 (99.82)	543 (0.18)		14,191 (99.85)	21 (0.15)	
	Current smoker	533,637 (99.69)	1,645 (0.31)		31,028 (99.76)	75 (0.24)	
Alcohol consumption				<0.001			0.224
	None	312,309 (99.71)	902 (0.29)		705,123 (99.88)	820 (0.12)	
	2-3 drinks/mo	548,255 (99.79)	1,146 (0.21)		236,485 (99.89)	257 (0.11)	
	1-4 drinks/wk	206,048 (99.77)	477 (0.23)		33,585 (99.88)	39 (0.12)	
	≥5 drinks/wk	68,622 (99.69)	214 (0.31)		11,510 (99.83)	20 (0.17)	
Exercise (times/wk)				<0.001			0.096
	No exercise	464,750 (99.74)	1,220 (0.26)		513,340 (99.88)	626 (0.12)	
	1-4	229,928 (99.77)	529 (0.23)		181,793 (99.89)	209 (0.11)	
	≥5	442,872 (99.78)	993 (0.22)		298,969 (99.90)	314 (0.10)	
Acute myocardial infarction				<0.001			<0.001
	Yes	5,335 (81.90)	1,179 (18.10)		599 (86.06)	97 (13.94)	
	No	1,141,544 (99.86)	1,584 (0.14)		1,000,196 (99.89)	1,062 (0.11)	
Family history of hypertension				0.031			0.012
	Yes	119,251 (99.74)	314 (0.26)		127,569 (99.87)	168 (0.13)	
	No	678,868 (99.77)	1,564 (0.23)		535,269 (99.89)	566 (0.11)	
Family history of diabetes mellitus				0.001			0.483
	Yes	109,954 (99.72)	308 (0.28)		102,395 (99.88)	120 (0.12)	
	No	687,487 (99.77)	1,571 (0.23)		560,104 (99.89)	612 (0.11)	
Family history of stroke				0.214			0.999
	Yes	85,003 (99.75)	217 (0.25)		65,377 (99.89)	72 (0.11)	
	No	713,089 (99.77)	1,664 (0.23)		596,696 (99.89)	657 (0.11)	
Total cholesterol (mg/dL)				<0.001			<0.001
	<200	601,702 (99.79)	1,268 (0.21)		597,109 (99.89)	631 (0.11)	
	200-239	399,277 (99.77)	940 (0.23)		307,422 (99.88)	383 (0.12)	
	>239	145,900 (99.62)	555 (0.38)		96,264 (99.85)	145 (0.15)	
ALT (IU/L)				<0.001			<0.001
	<40	919,272 (99.78)	2,070 (0.22)		952,491 (99.91)	1,048 (0.09)	
	40-99	210,218 (99.71)	607 (0.29)		43,668 (99.88)	337 (0.12)	
	>100	17,389 (99.51)	86 (0.49)		159,829 (99.86)	231 (0.14)	
Hemoglobin (g/dL)				<0.001			0.054
	<13.5 (male), <12.0 (female)	87,087 (99.68)	279 (0.32)		222,419 (99.88)	270 (0.12)	
	13.5-17.5 (male), 12.0-15.5 (female)	1,047,180 (99.77)	2,418 (0.23)		773,363 (99.89)	878 (0.11)	
	>17.5 (male), >15.5 (female)	12,612 (99.48)	66 (0.62)		4,929 (99.78)	11 (0.22)	
Creatinine (mg/dL)				<0.001			<0.001
	≤1.5	1,105,044 (99.77)	2,601 (0.23)		986,158 (99.89)	1,115 (0.11)	
	>1.5	41,764 (99.61)	162 (0.39)		14,582 (99.70)	44 (0.30)	<0.001
Body mass index (kg/m^2^)				<0.001			
	<18.5	16,995 (99.69)	52 (0.31)		25,396 (99.87)	32 (0.13)	
	18.5-22.9	357,675 (99.79)	763 (0.21)		487,723 (99.91)	433 (0.09)	
	23-24.9	325,142 (99.79)	680 (0.21)		240,123 (99.89)	269 (0.11)	
	25-29.9	412,217 (99.73)	1,117 (0.27)		218,619 (99.85)	339 (0.15)	
	≥30.0	34,850 (99.57)	151 (0.43)		28,934 (99.70)	86 (0.30)	

Values are presented as number (%). HF, heart failure; Pre-MetS, pre-metabolic syndrome; MetS, metabolic syndrome; ALT, alanine aminotransferase.

1 Using chi-square test.

**Table 3.** Risk of heart failure in male: Cox proportional hazard model

Variables		Non-adjusted HR (95% CI)	Multivariable HR (95% CI)			
		Model 1	Model 2	Model 3	Model 4	Model 5
Metabolic status						
	Normal	1.000 (reference)	1.000 (reference)	1.000 (reference)	1.000 (reference)	1.000 (reference)
	Pre-metabolic syndrome	1.867 (1.599, 2.180)^***^	1.846 (1.581, 2.156)^***^	1.839 (1.575, 2.148)^***^	1.778 (1.517, 2.083)^***^	1.508 (1.287, 1.767)^***^
	Metabolic syndrome	3.016 (2.569, 3.541)^***^	2.930 (2.494, 3.442)^***^	2.901 (2.469, 3.410)^***^	2.584 (2.167, 3.081)^***^	1.711 (1.433, 2.044)^***^
Age		-	1.067 (1.050, 1.084)^***^	1.067 (1.050, 1.085)^***^	1.069 (1.051, 1.086)^***^	1.034 (1.017, 1.051)^***^
Smoking status						
	Non-smoker	-	1.000 (reference)	1.000 (reference)	1.000 (reference)	1.000 (reference)
	Ex-smoker	-	0.994 (0.858, 1.151)	0.982 (0.848, 1.138)	0.971 (0.838, 1.125)	0.906 (0.782, 1.050)
	Current smoker	-	1.800 (1.592, 2.035)^***^	1.787 (1.580, 2.020)^***^	1.772 (1.567, 2.005)^***^	1.190 (1.051, 1.348)^**^
Alcohol consumption						
	None	-	1.000 (reference)	1.000 (reference)	1.000 (reference)	1.000 (reference)
	2-3 drinks/mo	-	0.640 (0.573, 0.714)^***^	0.638 (0.571, 0.712)^***^	0.643 (0.576, 0.718)^***^	0.814 (0.729, 0.909)^***^
	1-4 drinks/wk	-	0.666 (0.580, 0.765)^***^	0.665 (0.579, 0.764)^***^	0.674 (0.587, 0.774)^***^	0.975 (0.848, 1.120)
	≥5 drinks/wk	-	0.806 (0.667, 0.974)^*^	0.807 (0.668, 0.975)^*^	0.816 (0.675, 0.986)^*^	1.162 (0.961, 1.404)
Exercise (times/wk)						
	No exercise	-	1.000 (reference)	1.000 (reference)	1.000 (reference)	1.000 (reference)
	1-4	-	0.936 (0.826, 1.062)	0.933 (0.822, 1.058)	0.928 (0.818, 1.052)	0.927 (0.817, 1.051)
	≥5	-	0.964 (0.869, 1.069)	0.956 (0.862, 1.060)	0.954 (0.860, 1.059)	0.985 (0.888, 1.094)
Family history of heart disease						
	No	-	-	1.000 (reference)	1.000 (reference)	1.000 (reference)
	Yes	-	-	1.328 (1.108, 1.593)^**^	1.339 (1.117, 1.605)^**^	1.153 (0.964, 1.380)
Family history of hypertension						
	No	-	-	1.000 (reference)	1.000 (reference)	1.000 (reference)
	Yes	-	-	1.033 (0.904, 1.180)	1.042 (0.912, 1.189)	0.991 (0.869, 1.130)
Family history of diabetes mellitus						
	No	-	-	1.000 (reference)	1.000 (reference)	1.000 (reference)
	Yes	-	-	1.110 (0.972, 1.267)	1.116 (0.978, 1.274)	1.055 (0.926, 1.203)
Family history of stroke						
	No	-	-	1.000 (reference)	1.000 (reference)	1.000 (reference)
	Yes	-	-	0.985 (0.845, 1.148)	0.997 (0.855, 1.162)	0.982 (0.844, 1.143)
Body mass index (kg/m^2^)						
	<18.5	-	-	-	1.629 (1.154, 2.298)	1.786 (1.266, 2.520)^***^
	18.5-22.9	-	-	-	1.000 (reference)	1.000 (reference)
	23-24.9	-	-	-	0.926 (0.813, 1.053)	0.883 (0.776, 1.005)
	25-29.9	-	-	-	1.008 (0.890, 1.142)	0.929 (0.820, 1.052)
	≥30.0	-	-	-	1.340 (1.066, 1.686)^*^	1.223 (0.972, 1.540)
Hemoglobin (g/dL)						
	<13.5 (male), <12.0 (female)	-	-	-	1.498 (1.273, 1.763)^*^	1.553 (1.319, 1.829)^***^
	13.5-17.5 (male), 12.0-15.5 (female)	-	-	-	1.000 (reference)	1.000 (reference)
	>17.5, >15.5 (female)	-	-	-	1.446 (1.047, 1.999)^***^	1.081 (0.782, 1.494)
Creatinine (mg/dL)						
	≤1.5	-	-	-	1.000 (reference)	1.000 (reference)
	>1.5	-	-	-	1.735 (1.455, 2.068)^***^	1.624 (1.362, 1.937)^***^
Total cholesterol (mg/dL)						
	<200	-	-	-	1.000 (reference)	1.000 (reference)
	200-239	-	-	-	1.066 (0.960, 1.184)	0.888 (0.799, 0.986)^*^
	>239	-	-	-	1.606 (1.417, 1.821)^***^	0.974 (0.857, 1.107)
ALT (IU/L)						
	<40	-	-	-	1.000 (reference)	1.000 (reference)
	40-99	-	-	-	1.092 (0.973, 1.226)	1.082 (0.964, 1.214)
	≥100	-	-	-	1.436 (1.067, 1.933)^*^	1.591 (1.183, 2.139)^**^
Acute myocardial infarction^1^						
	No	-	-	-	-	1.000 (reference)
	Yes	-	-	-	-	130.854 (118.389, 144.631)^***^

HR, hazard ratio; CI, confidence interval; Pre-MetS, pre-metabolic syndrome; MetS, metabolic syndrome, ALT, alanine aminotransferase.

1 Occurrence of acute myocardial infarction during the follow-up period.

* p<0.05,

** p<0.01,

*** p<0.001.

**Table 4.** Risk of heart failure in female: Cox proportional hazard model.

Variables		Non-adjusted HR (95% CI)	Multivariable HR (95% CI)			
		Model 1	Model 2	Model 3	Model 4	Model 5
Metabolic status						
	Normal	1.000 (reference)	1.000 (reference)	1.000 (reference)	1.000 (reference)	1.000 (reference)
	Pre-metabolic syndrome	1.605 (1.340, 1.923)^***^	1.555 (1.298, 1.864)^***^	1.548 (1.291, 1.856)^***^	1.436 (1.193, 1.730)^***^	1.395 (1.158, 1.681)^***^
	Metabolic syndrome	3.349 (2.706, 4.145)^***^	3.118 (2.515, 3.866)^***^	3.086 (2.486, 3.830)^***^	2.324 (1.817, 2.973)^***^	2.144 (1.674, 2.747)^***^
Age		-	1.056 (1.029, 1.084)^***^	1.056 (1.029, 1.085)^***^	1.057 (1.029, 1.085)^***^	1.051 (1.023, 1.079)^***^
Smoking status						
	Non-smoker	-	1.000 (reference)	1.000 (reference)	1.000 (reference)	1.000 (reference)
	Ex-smoker	-	0.839 (0.434, 1.624)	0.838 (0.433, 1.622)	0.840 (0.434, 1.626)	0.847 (0.437, 1.639)
	Current smoker	-	1.990 (1.452, 2.726)^***^	1.990 (1.452, 2.727)^***^	1.995 (1.455, 2.735)^***^	1.721 (1.254, 2.362)^***^
Alcohol consumption						
	None	-				1.000 (reference)
	2-3 drinks/mo	-	0.942 (0.787, 1.127)	0.942 (0.787, 1.127)	0.952 (0.795, 1.140)	0.964 (0.805, 1.154)
	1-4 drinks/wk	-	0.909 (0.603, 1.370)	0.909 (0.603, 1.370)	0.924 (0.613, 1.392)	0.931 (0.618, 1.403)
	≥5 drinks/wk	-	1.353 (0.787, 2.324)	1.354 (0.788, 2.326)	1.363 (0.793, 2.341)	1.435 (0.837, 2.461)
Exercise (times/wk)						
	No exercise	-	1.000 (reference)	1.000 (reference)	1.000 (reference)	1.000 (reference)
	1-4	-	0.941 (0.769, 1.151)	0.938 (0.767, 1.148)	0.939 (0.767, 1.149)	0.943 (0.771, 1.155)
	≥5	-	0.898 (0.755, 1.066)	0.894 (0.752, 1.063)	0.901 (0.758, 1.070)	0.915 (0.770, 1.088)
Family history of heart disease						
	No	-	-	1.000 (reference)	1.000 (reference)	1.000 (reference)
	Yes	-	-	1.080 (0.792, 1.474)	1.088 (0.797, 1.484)	1.088 (0.798, 1.485)
Family history of hypertension						
	No	-	-	1.000 (reference)	1.000 (reference)	1.000 (reference)
	Yes	-	-	1.130 (0.942, 1.356)	1.126 (0.939, 1.351)	1.086 (0.905, 1.303)
Family history of diabetes mellitus						
	No	-	-	1.000 (reference)	1.000 (reference)	1.000 (reference)
	Yes	-	-	0.976 (0.795, 1.197)	0.972 (0.792, 1.193)	0.985 (0.802, 1.209)
Family history of stroke						
	No	-	-	1.000 (reference)	1.000 (reference)	1.000 (reference)
	Yes	-	-	0.966 (0.753, 1.238)	0.972 (0.758, 1.246)	0.974 (0.760, 1.249)
Body mass index (kg/m^2^)						
	<18.5	-	-	-	1.647 (1.043, 2.600)^*^	1.663 (1.053, 2.624)^*^
	18.5-22.9	-	-	-	1.000 (reference)	1.000 (reference)
	23-24.9	-	-	-	1.233 (1.014, 1.500)^*^	1.226 (1.008, 1.492)^*^
	25-29.9	-	-	-	1.305 (1.070, 1.593)^**^	1.273 (1.042, 1.554)^*^
	≥30.0	-	-	-	2.152 (1.571, 2.946)^***^	2.085 (1.523, 2.853)^***^
Hemoglobin (g/dL)						
	<13.5 (male), <12.0 (female)	-	-	-	1.207 (1.009, 1.444)^*^	1.203 (1.006, 1.439)^*^
	13.5-17.5 (male), 12.0-15.5 (female)	-	-	-	1.000 (reference)	1.000 (reference)
	>17.5 (male), >15.5 (female)	-	-	-	1.562 (0.775, 3.148)	1.496 (0.742, 3.015)
Creatinine (mg/dL)						
	≤1.5	-	-	-	1.000 (reference)	1.000 (reference)
	>1.5	-	-	-	2.300 (1.603, 3.298)^***^	2.167 (1.510, 3.109)^***^
Total cholesterol (mg/dL)						
	<200	-	-	-	1.000 (reference)	1.000 (reference)
	200-239	-	-	-	0.966 (0.818, 1.140)	0.977 (0.827, 1.153)
	>239	-	-	-	1.038 (0.818, 1.318)	1.011 (0.797, 1.283)
ALT (IU/L)						
	<40	-	-	-	1.000 (reference)	1.000 (reference)
	40-99	-	-	-	1.643 (1.265, 2.132)^***^	1.551 (1.193, 2.016)^***^
	≥100	-	-	-	1.942 (0.963, 3.914)	1.753 (0.869, 3.537)
Acute myocardial infarction^1^						
	No	-	-	-	-	1.000 (reference)
	Yes	-	-	-	-	82.637 (61.643, 110.781)^***^

HR, hazard ratio; CI, confidence interval; Pre-MetS, pre-metabolic syndrome; MetS, metabolic syndrome; ALT, alanine aminotransferase.

1 Occurrence of acute myocardial infarction during the follow-up period.

* p<0.05,

** p<0.01,

*** p<0.001.

**Table 5.** Risk of heart failure according to each component of metabolic syndrome: Cox proportional hazard model

Variables	Multivariable HR (95% CI)	
	Male	Female
Abdominal obesity	1.238 (1.087, 1.409)^**^	1.234 (0.973, 1.566)
Elevated blood pressure	1.244 (1.130, 1.369)^***^	1.372 (1.168, 1.612)^***^
Elevated fasting glucose	1.172 (1.066, 1.289)^***^	1.258 (1.066, 1.484)^**^
High triglycerides	1.035 (0.935, 1.146)	1.020 (0.837, 1.242)
Low high‐density lipoprotein cholesterol	1.048 (0.929, 1.183)	1.159 (0.980, 1.371)

HR, hazard ratio; CI, confidence interval.

** p<0.01,

*** p<0.001.

## References

[1] Bozkurt B, Coats AJ, Tsutsui H, Abdelhamid M, Adamopoulos S, Albert N (2021). Universal definition and classification of heart failure: a report of the Heart Failure Society of America, Heart Failure Association of the European Society of Cardiology, Japanese Heart Failure Society and Writing Committee of the Universal Definition of Heart Failure. J Card Fail.

[2] Braunwald E (2013). Heart failure. JACC Heart Fail.

[3] Askoxylakis V, Thieke C, Pleger ST, Most P, Tanner J, Lindel K (2010). Long-term survival of cancer patients compared to heart failure and stroke: a systematic review. BMC Cancer.

[4] Park JJ, Lee CJ, Park SJ, Choi JO, Choi S, Park SM (2021). Heart failure statistics in Korea, 2020: a report from the Korean Society of Heart Failure. Int J Heart Fail.

[5] Aguilar M, Bhuket T, Torres S, Liu B, Wong RJ (2015). Prevalence of the metabolic syndrome in the United States, 2003–2012. JAMA.

[6] Gao W, DECODE Study Group (2008). Does the constellation of risk factors with and without abdominal adiposity associate with different cardiovascular mortality risk?. Int J Obes (Lond).

[7] Rochlani Y, Pothineni NV, Kovelamudi S, Mehta JL (2017). Metabolic syndrome: pathophysiology, management, and modulation by natural compounds. Ther Adv Cardiovasc Dis.

[8] Miura Y, Fukumoto Y, Shiba N, Miura T, Shimada K, Iwama Y (2010). Prevalence and clinical implication of metabolic syndrome in chronic heart failure. Circ J.

[9] Li C, Ford ES, McGuire LC, Mokdad AH (2007). Association of metabolic syndrome and insulin resistance with congestive heart failure: findings from the Third National Health and Nutrition Examination Survey. J Epidemiol Community Health.

[10] Tamariz L, Hassan B, Palacio A, Arcement L, Horswell R, Hebert K (2009). Metabolic syndrome increases mortality in heart failure. Clin Cardiol.

[11] Perrone-Filardi P, Savarese G, Scarano M, Cavazzina R, Trimarco B, Minneci S (2015). Prognostic impact of metabolic syndrome in patients with chronic heart failure: data from GISSI-HF trial. Int J Cardiol.

[12] Koutroumpakis E, Jozwik B, Aguilar D, Taegtmeyer H (2020). Strategies of unloading the failing heart from metabolic stress. Am J Med.

[13] Ingelsson E, Arnlöv J, Lind L, Sundström J (2006). Metabolic syndrome and risk for heart failure in middle-aged men. Heart.

[14] Bahrami H, Bluemke DA, Kronmal R, Bertoni AG, Lloyd-Jones DM, Shahar E (2008). Novel metabolic risk factors for incident heart failure and their relationship with obesity: the MESA (Multi-Ethnic Study of Atherosclerosis) study. J Am Coll Cardiol.

[15] Butler J, Rodondi N, Zhu Y, Figaro K, Fazio S, Vaughan DE (2006). Metabolic syndrome and the risk of cardiovascular disease in older adults. J Am Coll Cardiol.

[16] Wang J, Sarnola K, Ruotsalainen S, Moilanen L, Lepistö P, Laakso M (2010). The metabolic syndrome predicts incident congestive heart failure: a 20-year follow-up study of elderly Finns. Atherosclerosis.

[17] Ho JE, Lyass A, Lee DS, Vasan RS, Kannel WB, Larson MG (2013). Predictors of new-onset heart failure: differences in preserved versus reduced ejection fraction. Circ Heart Fail.

[18] Ho JE, Gona P, Pencina MJ, Tu JV, Austin PC, Vasan RS (2012). Discriminating clinical features of heart failure with preserved vs. reduced ejection fraction in the community. Eur Heart J.

[19] Regitz-Zagrosek V (2020). Sex and gender differences in heart failure. Int J Heart Fail.

[20] Simmonds SJ, Cuijpers I, Heymans S, Jones EA (2020). Cellular and molecular differences between HFpEF and HFrEF: a step ahead in an improved pathological understanding. Cells.

[21] Sulo G, Igland J, Vollset SE, Nygård O, Ebbing M, Sulo E (2016). Heart failure complicating acute myocardial infarction; burden and timing of occurrence: a nation-wide analysis including 86 771 patients from the Cardiovascular Disease in Norway (CVDNOR) Project. J Am Heart Assoc.

[22] He J, Ogden LG, Bazzano LA, Vupputuri S, Loria C, Whelton PK (2001). Risk factors for congestive heart failure in US men and women: NHANES I epidemiologic follow-up study. Arch Intern Med.

[23] Levy D, Larson MG, Vasan RS, Kannel WB, Ho KK (1996). The progression from hypertension to congestive heart failure. JAMA.

[24] Kenchaiah S, Evans JC, Levy D, Wilson PW, Benjamin EJ, Larson MG (2002). Obesity and the risk of heart failure. N Engl J Med.

[25] Savji N, Meijers WC, Bartz TM, Bhambhani V, Cushman M, Nayor M (2018). The association of obesity and cardiometabolic traits with incident HFpEF and HFrEF. JACC Heart Fail.

[26] Eaton CB, Pettinger M, Rossouw J, Martin LW, Foraker R, Quddus A (2016). Risk factors for incident hospitalized heart failure with preserved versus reduced ejection fraction in a multiracial cohort of postmenopausal women. Circ Heart Fail.

[27] Huffman MD, Berry JD, Ning H, Dyer AR, Garside DB, Cai X (2013). Lifetime risk for heart failure among white and black Americans: cardiovascular lifetime risk pooling project. J Am Coll Cardiol.

[28] Björck L, Lundberg C, Schaufelberger M, Lissner L, Adiels M, Rosengren A (2020). Body mass index in women aged 18 to 45 and subsequent risk of heart failure. Eur J Prev Cardiol.

[29] Rosengren A, Åberg M, Robertson J, Waern M, Schaufelberger M, Kuhn G (2017). Body weight in adolescence and long-term risk of early heart failure in adulthood among men in Sweden. Eur Heart J.

[30] Rhee EJ, Kwon H, Park SE, Han KD, Park YG, Kim YH (2020). Associations among obesity degree, glycemic status, and risk of heart failure in 9,720,220 Korean adults. Diabetes Metab J.

[31] Varat MA, Adolph RJ, Fowler NO (1972). Cardiovascular effects of anemia. Am Heart J.

[32] Klip IT, Postmus D, Voors AA, Brouwers FP, Gansevoort RT, Bakker SJ (2015). Hemoglobin levels and new-onset heart failure in the community. Am Heart J.

[33] Coglianese EE, Qureshi MM, Vasan RS, Wang TJ, Moore LL (2012). Usefulness of the blood hematocrit level to predict development of heart failure in a community. Am J Cardiol.

